# Lifestyle changes after cancer treatment in patients and their partners: a qualitative study

**DOI:** 10.1007/s00520-024-08447-w

**Published:** 2024-03-26

**Authors:** Marrit Annika Tuinman, Janine Nuver, Anke de Boer, Anne Looijmans, Mariët Hagedoorn

**Affiliations:** 1 Department of Health Psychology, University Medical Center Groningen, University of Groningen, Hanzeplein 1, 9713 GZ Groningen, The Netherlands; 2 Department of Medical Oncology, University Medical Center Groningen, University of Groningen, Hanzeplein 1, 9713 GZ Groningen, The Netherlands; 3 Department of Psychology, Patyna Elderly Care, Harste 15, 8602 JX Sneek, The Netherlands

**Keywords:** Testicular cancer, Breast cancer, Lifestyle, Spouses, Partners, Health behavior

## Abstract

**Purpose:**

Oncologists nowadays promote healthy lifestyle choices more often, focusing on diet, physical activity, smoking, alcohol consumption, and sleep, but the question is whether this is enough to establish actual change. As patients will have to achieve a healthy lifestyle at home in daily life, it is important to understand barriers and facilitators for lifestyle change for both patients and their partners.

**Methods:**

A qualitative interview study was done among patients who received chemotherapy for testicular (*n* = 10) or breast cancer (*n* = 7) and their partners (*n* = 17). The interview focused on how much they remembered the lifestyle advice given in hospital, whether and what they had adapted since diagnosis, and what they deemed as facilitators and barriers in maintaining lifestyle change.

**Results:**

Results showed that many patients and partners recalled that some advice was given in hospital but experienced this as too general and only at the start of treatment. Social contacts and the entire cancer experience helped facilitate change but were also seen as barriers. Other barriers were not considering healthy behaviors a priority or experiencing unhealthy choices as something nice after a trying time.

**Conclusions:**

Oncologists and hospitals that provide lifestyle advice should provide cancer- and person-specific lifestyle advice, should offer this advice repeatedly into survivorship, and include the partner, as they are dedicated to improving lifestyle as well.

**Implication for cancer survivors:**

Staying healthy after cancer is important to both patients and their partners, and both experience their own facilitators and barriers to achieving this. Seeing a healthy lifestyle as a joint goal might facilitate change.

Lifestyle plays an important role throughout the cancer continuum: from etiology to treatment efficacy, treatment toxicity, rehabilitation, recurrence risk, cancer-specific, and overall survival. Several observational studies have shown the importance of lifestyle factors, like physical activity and a healthy diet, for survival and recurrence after cancer treatment [1, 2]. Various reports suggest that physical activity after a cancer diagnosis is associated with better cancer-specific and overall survival in individuals diagnosed with breast, colorectal, or prostate cancer [3]. The same holds for smoking cessation, which is related to fewer secondary malignancies after a first diagnosis and better survival after several types of cancer, including types other than the expected smoking-related diagnoses, such as lung or head and neck cancers [4]. Moreover, a healthy lifestyle can improve quality of life and reduce fatigue in patients after treatment for various cancer types [5–7].

Following cancer treatment, albeit related differently to different treatment modalities, an increased risk of cardiovascular disease (CVD) and type 2 diabetes mellitus is common [8]. During the first 5 years of follow-up after platinum-based chemotherapy, survivors of testicular cancer frequently develop risk factors for CVD, like overweight, hypertension, and hypercholesterolemia [9]; more than 10 years after chemotherapy, these patients have an increased risk of myocardial infarction, compared with men from the general population [10]. Following anthracycline-containing adjuvant chemotherapy, the risk of heart failure is increased in breast cancer survivors [11], as is the risk of weight gain after treatment completion [12]. Equal or even higher prevalence of CVD risk factors has been reported for survivors of prostate, colorectal, and gynecologic cancers (4–14 years after diagnosis), compared to the general adult population in a cross-sectional survey [13]. These data underscore the importance of rigorous CVD risk factor management and, thus, healthy lifestyle counseling for cancer survivors following treatment.

Based on increasing awareness of cancer-treatment-related toxicity and a general growing insight into healthy behaviors, specific nutrition and physical activity guidelines for patients with cancer have been developed [14]. These guidelines advise achieving and maintaining a healthy weight by eating unprocessed foods that are high in fiber, not drinking alcohol (or at least limiting it to one drink per day), and being physically active for at least 150 min per week. Oncologists acknowledge the need to advise their patients on a healthy lifestyle, but do not necessarily follow up on this need, and sometimes do not know how to provide adequate advice [15]. They experience certain barriers to providing lifestyle guidance, such as lack of time, lack of guidelines, or hesitations due to fearing loss of connection with their patients by bringing up health behaviors. However, the vast majority of both patients with cancer and the people in their social network see advice on diet, weight, and physical activity as beneficial. They also see their doctor as the right person to provide this, and only a few feel that this advice would be insensitive or blame the patient [16]. Patients themselves also reported that they knew about the benefits of a healthy diet and were motivated to know more, but that they did not receive professional advice about this [17].

Previous studies on cancer survivors have reported that, even though they are at a higher risk for long-term health consequences, rates of unhealthy lifestyle behaviors are similar to the general population [18]. In young adult patients with cancer, worse health behaviors were also reported, as women tended to smoke more often than their peers, and men ate less fruit and vegetables [19]. Furthermore, a cancer diagnosis in itself is not enough to motivate durable beneficial health behavior change in cancer survivors [20]. In order to actually instigate lifestyle changes, cancer patients need an environment that is supportive. Social support, in particular support from their spouse or partner, may have an important impact on the motivation and ability of the patient with cancer to change his or her lifestyle. For example, attempts to increase exercise or stop smoking may be more successful and durable if a partner is supportive or shows the same behavior [21]. After prostate cancer, an involved and supportive partner can increase adherence to a pelvic floor exercise program [22]. Furthermore, cancer patients’ and partners’ health behavior was found to be highly comparable with respect to physical activity, and eating fruit and vegetables [23]. When patients and partners were more equal in their health behaviors, they were also more satisfied with the relationship and experienced their partner as being more supportive.

For partners to have a beneficial influence on the patients’ health behaviors, they also need to lead a healthy lifestyle. However, as is the case for patients themselves, a cancer diagnosis is not always enough for partners to change their health behavior in the long term. Family members of cancer patients who were completing treatment became more aware of the importance of a healthy lifestyle due to their confrontation with cancer but indicated less motivation for making actual changes [24]. A review on health behavior change among caregivers of patients with cancer revealed mixed results; some studies showed improvement, while others showed deteriorated health behaviors [25]. The same was found in a large study among Danish partners of patients with cancer. Partners did adapt their lifestyle, but only for the better if the patient had a good prognosis and was still alive [26]. In short, patients with cancer should get advice on a healthy lifestyle following cancer but seem to have similar lifestyle behaviors as people without a cancer history. Also, health behaviors of patients and partners seem to be correlated, and having a supportive partner helps to adopt a healthy lifestyle, but partners struggle to adapt behavior themselves. Therefore, further insight is needed into the barriers to and facilitators of a healthy lifestyle in patients as well as their partners and how to promote healthy behavior after a cancer diagnosis.

The current qualitative study with semi-structured interviews among breast and testicular cancer patients and their partners provides more insight into how couples handle lifestyle following the diagnosis of cancer and enables improvements in lifestyle counseling in hospital. We examined what patients and partners remembered about the lifestyle advice given in hospital, which major lifestyle behaviors they adapted, and what they perceived as facilitating factors or barriers to achieve change.

## Methods

According to Dutch law regarding medical research involving human subjects (WMO), the Medical Ethical Committee of the University Medical Center Groningen of Groningen provided a waiver for ethical assessment (METc 2015/495). The study was conducted according to the Declaration of Helsinki and Good Clinical Practice Guidelines. This paper is written according to the reporting guidelines of O’Brien et al. [27].

### Approach

We employed a qualitative approach, conducting semi-structured interviews to collect data. Firstly, participants were asked to recall which advice about a healthy lifestyle they received from health care professionals in hospital during treatment and follow-up. Secondly, participants chose from a list a health behavior they adapted since diagnosis (i.e., alcohol intake, smoking, diet, physical activity, or sleep/relaxation) or, if not on that list, they could specify a different one. Thereafter, they were asked about their experiences in changing this behavior, what helped them to maintain this change (facilitators), and what made it harder to (maintain) change (barriers). Patients and partners who did not change anything in their health behavior were asked about which barriers they perceived to make a change.

### Participants and procedure

Patients with a diagnosis of either breast or testicular cancer, who had received curative chemotherapy at the Department of Medical Oncology of the University Medical Center Groningen, the Netherlands, and their partners were invited to participate. By inviting patients with these diagnoses, we aimed to recruit an equal sample of both female and male patients, to account for possible gender differences in lifestyle behavior. The inclusion criteria were (1) chemotherapy was completed no longer than 24 months ago; adjuvant hormone therapy for breast cancer was allowed, (2) the patient had no signs of metastases/ a good prognosis, (3) the patient was in active follow-up, (4) the patient was in a committed relationship and sharing a household with a partner, (5) the patient and partner had to be able to speak Dutch, and (6) the patient and partner had to provide written informed consent. JN (Medical Oncologist, MD PhD) screened the complete patient list of the patients she treated during the previous 2 years for eligibility. Thereafter, testicular cancer patients and partners were invited consecutively by JN during a follow-up visit to the outpatient clinic, in which they received a package with an information letter explaining the study, informed consent forms, and a prepaid return envelope. JN invited breast cancer patients and their partners by mail with the same package. Of the testicular cancer patients and partners, 10 of the 20 couples that were invited participated (response rate 50%). Of the 17 breast cancer patients and partners that were invited, seven couples participated (response rate 41%).

Patients and partners who both returned their own informed consent forms were invited by phone for an interview to be scheduled following a hospital appointment to avoid additional travel. Interviews took place in 2016 and 2017. Patients were interviewed first in an office at the outpatient clinic or at the Department of Health Psychology. Partners received a coupon to get a coffee or tea while waiting, and the same was offered to patients while the partners were interviewed. The interviews were conducted by MT (post-doc in Health Psychology, PhD), who was trained in qualitative research methods, and a student assistant of the Bachelor Applied Psychology (AdB, BSc), who was trained by MT. The interview time varied between 22 and 44 min. The interviews were recorded and transcribed verbatim by the student assistant. The quality of the transcripts was checked by MT, by randomly examining 10 time points per recording, and thereafter imported into ATLAS.ti V7.

### Analyses

For reliability, we employed data source and investigator triangulation. Data source triangulation was obtained by interviewing both patients and partners on their own experiences to obtain both perspectives. We considered patients and partners to be equal, but separate units of analysis. Patients and partners were both asked about what they remembered from the advice given to the patient in the hospital. Thereafter, the interview focused on their own lifestyle changes, facilitators, and barriers. To reach investigator triangulation, interviews, data checking, and coding were done by varying teams, including researchers not involved in patient care (MT, MH (professor of Health Psychology, PhD) and AL (post-doc in Health Psychology, PhD)) and researchers not involved in the interview process (JN and AL).

First, all the transcripts were coded by MT and an independent qualitative researcher of the Department of Applied Health Sciences at the University Medical Center Groningen to organize all the quotes into the interview question categories (advice received, behavior changed, facilitators, and barriers). Thematic analysis approach within each interview question was used to identify patterns of codes (themes) [28]. MT and the independent researcher proceeded by coding the themes within the question categories “advice received,” “behavior changed,” and “facilitators.” Themes within the question category “barriers” were coded by MT, JN, and MH. Following each cycle of independently coding 2 or 3 transcripts, the codes were discussed until agreement was reached, and the coding schedule was adapted or expanded accordingly. The subsequent transcripts were coded with the updated coding schedule.

## Results

### Sample

The ten patients (males) with testicular cancer (TC) were on average 38 years of age at the time of interview (range 30–45 years), and the seven patients (females) with breast cancer (BC) were on average 62 years of age (range 52–74 years). Partners did not provide their birth date, so we could not indicate their age, but no large age gaps were observed or indicated during the interviews. One patient with testicular cancer had a male partner; the other patients were in a heterosexual relationship.

### Advice received from health care professionals in hospital

Ten patients (59%) and seven partners (41%) indicated they received some advice regarding lifestyle behaviors in hospital, and they remembered that this happened during active treatment, often via a brochure or folder. At that moment, lifestyle advice was mostly set aside, apart from the advice directly related to chemotherapy.

Patient with BC 4: “*I have to spit deeply into my memory…. I can’t remember, or it didn’t impress me much. You’re so busy with other stuff, of course, and with chemotherapy, you can’t do certain things, but I set that aside since then*.”

Some patients and one partner also indicated that during treatment, the hospital should stick to its own advice. With respect to sleep/rest and diet, they indicated that the care in hospital sometimes contradicted the advice given, for example, the lack of a calm environment in the patient rooms and offering meals with lots of dairy products and animal fats. Five patients and a partner indicated that after the treatment was completed, all advice ceased regarding health behaviors. Some said that the advice changed when the patient was no longer receiving treatment.

Patient with TC 7: “… I believe advice would have impact, but it just didn’t happen. The doctors focus on getting you well again and after that you’re thrown back into sea so to speak.”

Partner of patient with TC 7: “Well, while he was ill, they were happy if he would eat during chemo. Like in, just eat whatever you can, even if it is unhealthy. Just don’t lose weight. But after treatment, his belly grew, you know, what men have. And after talking to the oncologist, he [patient] said, ok, so now I have to transition from eating everything to losing weight, ok, fine.”

Almost all patients and partners indicated they forgot the details of the advice given, and many also indicated that the advice was not specific enough or that it did not stick.

Partner of patient with TC 6: “Yes of course we got brochures, and they were easy to read. But, after saying you should do this or that, they don’t tell you how you should do that. For example, eat a healthy diet. What is healthy? … And for sleep, how many hours is good? For healthy people 6 to 9 hours, but what about cancer patients? From how many hours on should I be worried and think he’s slowly dying now?”.

Some patients and two partners felt that the advice given did not apply to them as they already led a healthy lifestyle. Some others indicated that, due to the advice not being specific, they turned to a care professional outside the hospital, for example, a dietician, a rehabilitation doctor, or a physical therapist for exercise, and indicated they had to take initiative for this themselves.

### Behavior change after diagnosis

Patients changed their behavior with respect to their diet (seven patients with TC and one with BC), followed by smoking (four patients with TC and two patients with BC), exercise (three TC patients), sleep or rest (one patient with TC and three with BC), enjoying life more/reducing stress (two patients with BC), drinking less alcohol (one patient with TC), or nothing (one patient with BC). Patients sometimes regarded their behavior change as more of a total package, in which they tweaked certain aspects simultaneously. Others indicated that they were already living quite healthily. With respect to smoking, besides quitting, some patients also adapted their behavior by reducing the number of cigarettes they smoked, started smoking outside, or started using an e-cigarette. Some patients mentioned that they became aware that certain behaviors are considered a risk factor for developing cancer, and referred to that when explaining why they changed a specific behavior.

Patient with BC 4: “Before I was diagnosed, I never really grasped that alcohol can cause cancer. But now I’m worried for my life, I mean, it’s alcohol, so sure, I will drink it sometimes and that will not be a big issue. But daily drinking, no, I don’t believe in that anymore, I don’t think that is right.”

Patient with TC 4: “I once read that sugars can contribute to getting cancer, so I’m really trying to change that. …. At least, no refined sugars, only natural ones.”

Patients and partners did not always match in the type of behavior change they made. Partners indicated that they changed their diet (six female partners), smoking (one male and two female partners), enjoying life/taking more rest (two male and two female partners), exercising (one male and one female partner) but also changing nothing (three male partners). Partners were more unidimensional in their changes as compared to patients, mentioning only one aspect, and more often indicated changing nothing since diagnosis. After probing, these partners eventually did indicate some changes, for example, that there was more time for, or focus on exercise after diagnosis, or that they now focused more on enjoying life and making time for relaxation. Female partners of testicular cancer patients often mentioned having changed their diet. Even though it was a personal change, for some, the underlying reason was the health of their husbands. Most of these partners were the main organizers of the home-cooked meals, and they also became more directive in whether take-out meals were acceptable. One partner changed their diet to accommodate the salt-free diet her husband needed to follow due to renal failure after chemotherapy. Another partner indicated that because of the fertility challenges her husband now faced due to orchiectomy, she wanted to reach a healthy weight to support IVF. Even though patients indicated quitting smoking entirely, for partners, this appeared more difficult.

Partner of patient with TC 2: “I used to smoke way more, before diagnosis. I was planning on quitting, but to be honest I do smoke sometimes, in secret.”

Partner of patient with BC 6: “But of course, because she is now a cancer patient and …. well, she quit. And then I said: you quit smoking, I will go outside, also during winter.”

### Facilitators to changing health behaviors

Patients and partners were asked what helped them in changing their behavior and maintaining that change. The most important themes that emerged were the confrontation with cancer, social and relationship factors, noticing the effects of behavior change, and their character.

Patients and partners both indicated that they were motivated to stay healthy and to prevent getting cancer (again) or prevent comorbidity.

Partner of patient with TC 2: “You see, you will never know why he got what he got, so you’re thinking to yourself, could it be related to food? And you’re trying to even out anything that could be related. You never know, but one thing is for sure, that with a healthy diet, well, and eating properly, you could stay healthy.”

Patient with BC 3: “I think, because of the cancer I get overweight quickly, so I have to avoid that. Because of overweight, I burden my body, my muscles, my joints. And I could get something else more easily, and I’m really aware of that.”

Patients and partners both experienced beneficial social factors in their wider social environment, as well as in their relationships. Patients, for example, mentioned that the support from the gym really motivated them to stay active even though they had trouble finding the energy to go, or that family members complimented them on the behavior change while they found change difficult.

Patient with TC 8: “Yeah, it helps. Indeed, people around us, in the family… There have been a few that tried to quit [smoking], but failed and they say to me that they think I did a good job and that I did succeed.”

With respect to the relationship factors, patients mentioned the role their partner (may have) had in their lifestyle change only when prompted, even though partners mentioned it spontaneously.

Interviewer: “You told me she picked up the cooking and dietary changes, what do you think about that?”.

Patient with TC 8: “Well, she’s fully supportive [by adapting the meals]. I mean, we know each other for thirty years, so… we know each other through and through. So it’s more of a non-discussed issue, it’s not like I have to say everyday ‘gosh, this is so great,’ that’s not how it is in our relationship.”

Partners mentioned the patient had motivated them to change, directly or indirectly. A female partner of a patient with TC indicated that the patient being so active after chemotherapy motivated her to become more active as well. Avoiding disappointment from the patient was also cited as an important facilitator.

Partner of patient with TC 7: “I thought, you went through all that cancer stuff, and of course, I dealt with it too, but not the chemo. Then who am I to nag about a year in my life, or about losing weight. If you can do it, I can do it. And I want to have the feeling like at least I tried everything I could to grow as old as possible together.”

Some partners mentioned their own role as important in changing behavior and making patients feel like the change was a joint effort, even though none of the patients mentioned them as facilitators. Partners indicated they were encouraging the patient to be active despite fatigue, or that they were there to jump in when the patient did not have the energy left to keep up the healthy behavior.

Partner of patient with TC 1: “Well, I really noticed that it is so important… the patient can’t do it just by himself. If you want to change your lifestyle, you have to focus on the family, on the partner. … You can only do it together, you know, you eat together, cook together, you watch TV and snack together, you can’t do all that alone. So you need the whole family.”

For patients, especially seeing the results of their efforts was a facilitator to go on. They mentioned seeing their weight decrease on the scale, getting positive feedback from the physicians based on their blood sample or blood pressure, or noticing better physical functioning. Patients and partners both mentioned that changing made them feel better.

Patient with TC 3: “and of course the kick is in hearing about the checkups that it has an effect, that’s of course… we really like that. And…”.

Interviewer: “so your wife enjoys that as well?”.

Patient: “yes, of course!”.

Patients often mentioned that their character helped them to maintain their changed behavior and that they have a mindset that helped them make changes. Some mentioned that they set certain rules or goals to avoid lapsing or being tempted to remain in unwanted behaviors.

Patient with TC 4: “Just don’t buy candy, just say that to yourself, don’t do it. There, done. It’s more intrinsic, it’s not that I need extra motivation or something like ‘you can’t have sugar’, no. I think people around me also know that about me, they know I take care of myself.”

Patient with BC 6: “I wanted to get through surgery as best as possible. And I’m… I have such strong discipline, it’s something I really learned from all this.”

### Barriers to changing health behaviors

Patients and partners also indicated what made changing and maintaining change more difficult for them. The most important barriers were not feeling motivated or making it a priority, unhealthy behaviors being nice/a reward, negative social factors, and long-term treatment-related effects. For motivation, the reasons were quite similar for patients and partners, they both indicated they needed to like something in order to keep going and often indicated that, for example, exercising was boring or stupid. With respect to drinking alcohol, eating unwanted foods, or smoking, both patients and partners indicated that this simply feels nice, that it is a social thing to do, and some of them even saw it as a reward to compensate for the cancer treatment period.

Patient with TC 7: “Yes, well you’re in sort of an ‘I don’t really care mode’ regarding food. When you have appetite again, when you can go out to dinner again and enjoy food, you’re like, I’m well again, what the heck, I’m going to dig in [laughs].”

Interviewer: “so that is important at such a moment?”.

Patient: “Yes, there’s a sort of compensation there, where you get sort of rebellious, or distant from your own…. Well, of course, I know what is better for me, but I just don’t want that right now. I’ll see about the rest later.”

Patient with BC 6: “For a long time, food didn’t taste nice, I just couldn’t taste flavors anymore. Then it is easy to not eat. But now that it’s better again, I picked up my old eating habits.”

Social factors were not only mentioned as facilitators of change but also as barriers, by both patients and partners, for example, having smokers or drinkers around, getting critical responses from others, the partner or family not changing their own behaviors, or patients feeling self-conscious about setting their own rules (for diet for example).

Patient with BC 3: “I understand and I don’t mind. But they can’t place themselves in my position, they think it’s overdone. They think I’m overreacting with my body. My family even more so than his [partner].”

Some patients also mentioned long-term treatment effects as a barrier to change or start a new behavior, for example, due to a lack of energy, decreased muscle strength, or having to deal with lymphedema.

Patient with BC 5: “And exercising too, I try to be active each day, whether it’s gardening or biking to work. Look and I just can’t … I used to bike every day, to and from work and I could manage going to the gym at night. But not anymore, and I don’t know if I will ever be able to do so.”

Patients mentioned barriers such as being too busy, experiencing a lack of specific advice or guidance, or feeling that healthy behaviors are difficult or hard to keep up. Patients also mentioned that their outlook on life and what is important shifted.

Patient with TC 7: “I finished my chemotherapy and I’m happy to still be here tomorrow. And… my decisions fit that feeling, I’m not busy with my future right now.”

## Discussion

This qualitative interview study with seventeen patients and their partners after treatment for breast or testicular cancer showed that the experience with cancer impacted their view on a healthy lifestyle and prompted almost all of them to make changes in their health behaviors. Patients and partners made dietary changes most often, followed by reducing or quitting smoking. More than half of the patients, and 41% of partners remembered getting some advice on a healthy lifestyle in the hospital, but this did not feel specific or personal enough, and the information was not repeated during survivorship. This advice was therefore not the crucial facilitator to initiate and maintain change. Several other facilitators and barriers to change were mentioned, with some noticeable differences between patients and partners.

Patients and partners remembered getting advice on adopting a healthy lifestyle, but it felt unpersonal and was not repeated into survivorship. This finding was also found in an interview study among survivors focusing on activity and diet only [29]. These survivors described a lack of advice and support following treatment completion, just as the couples in the current study. The same was found in an interview study with care professionals, patients, and family members on smoking cessation after cancer [30]. The care professionals indicated they asked whether patients smoked, but rarely followed up on this with advice or support for cessation. It is necessary to integrate lifestyle advice into the care pathway, with ample follow-up into the survivorship phase. While early lifestyle intervention, sometimes even before surgery, is found to be beneficial, interview studies also indicate barriers to change in the early phase of treatment and survivorship [31]. For example, the study on smoking indicated that family members would have been reluctant to quit smoking if it had been advised during treatment, because they had to deal with their own stress, and smoking was used as a way of helping to cope [30]. This also fits our finding that patients and partners mentioned that the experience with cancer made a healthy lifestyle less of a priority, a finding also supported by the study on diet and exercise where survivors reported a drop in motivation to change [29]. Our group emphasized that they wanted to give themselves a break after a stressful time and that unhealthy choices sometimes felt like a reward. On the other hand, having been confronted with cancer was also mentioned as a facilitator for change. The diagnosis and treatment of cancer made patients and partners aware of their motivation to remain healthy and to avoid long-term negative effects. They wanted to live a healthy life for as long as possible, but the cancer also made them focus on enjoying life in the here and now, making lifestyle changes less important.

These findings show that patients and partners need to be at the right time and level of motivation to change and that this right time is dependent on the treatment or follow-up schedule and the prevailing emotions. Attention for lifestyle change and offers for advice or support could therefore at best be repeated along the follow-up care, to fit with the patient’s and partner’s willingness and ability to change.

Besides repeating the offer of advice and support, patients and partners also indicated a need for personalized and more specific advice, as general guidelines and information did not feel fitting to them. The same was found in the study regarding activity and diet, where survivors felt that advice did not take into account the sometimes huge effects of cancer and its treatment (29). These survivors indicated that seeing very successful role models in health-promoting materials was intimidating and they felt that the difficulties cancer gave them were not acknowledged. The late effects of cancer were also mentioned as a barrier to change in our group, for example, being too fatigued to exercise, or dealing with lymphedema. Generic information is not tailored enough for a healthy lifestyle change after cancer. Receiving general advice, for example, provided by the Netherlands Nutrition Center, or reading general lifestyle brochures felt mismatched and did not fit with what patients and partners felt was relevant for them and their medical situation. That tailoring is difficult and was also reported in an interview study with prostate cancer patients, their partners, and their health care professionals [32]. These patients and partners indicated a need for fitting advice regarding their diet and physical activity, and most of the interviewed care professionals were aware that lifestyle interventions need to be tailored to the individual. This did not result in tailored advice, however, especially regarding advice on diet. The health care professionals indicated a lack of their own knowledge, as well as a lack of scientific dietary evidence. Lifestyle advice should, therefore, not only be offered repeatedly into survivorship but also match the specific needs and situation of the cancer patient and partner. It may be necessary to turn to cancer-specific sources or involve an oncology dietician or physical therapist in the follow-up care pathway to offer this.

Advice in hospital may be designed such that it is repeatedly offered, well-timed, and individually tailored, but patients and partners still have to achieve change at home, in their daily lives. The social surrounding was mentioned by patients and partners as having two sides, being both a facilitator and a barrier. Patients and partners both mentioned their larger social surroundings as important in whether the change was more or less difficult to reach. Family members and friends offered compliments, but also criticism. Especially when the lifestyle change after cancer did not match that of friends or family (anymore), maintaining the change was difficult, or felt socially uncomfortable. Probably the most influential social factor is the partner. The patients described the importance of their partner in changing more pragmatically, such as preparing unsalted meals, because the partner was always the one making dinner. However, in interviews with the patients, the importance of the partner had to be prompted. Partners themselves mentioned their own role in supporting the patient explicitly and spontaneously, indicating that they see their own role of importance in achieving change. Partners also saw the patient and what they dealt with as a motivator to positively change their own behavior, as an inspiration. This noticeable difference between recognizing the role of the partner in lifestyle change may have to do with the tension that exists between the patient wanting to remain independent and in control (especially after having been treated for cancer) and seeing and accepting support from the partner. For example, after prostate cancer, some patients experienced difficulty in doing their daily activities and switched between being independent and needing support to manage the impact of the illness and the side effects of the treatment [33].

In our study, patients mentioned their character as an important factor in reaching and maintaining change, indicating that their decision to change and their mindset were the important facilitators. This may be a sign of a need to feel in control again and may be opposite to the role partners see for themselves in supporting the lifestyle change. Following prostatectomy, patients and partners were found to better match in the desired amount of support when the patient’s independence and control capacity was larger [34]. This tension between providing support by partners and remaining independent as a patient is probably a factor that should be taken into account when lifestyle change is advised in the hospital.

Involving the partner may also meet other obstacles as seen in other studies. Involving the partner in planning how, when, and where to perform new behavior such as pelvic floor exercise has been found useful in couples coping with prostate cancer, but only when the patient is in the maintenance phase and not at the start of integrating this exercise into daily life [35]. Patients have also been found to feel reluctant to involve their partner in a couple-based exercise program, not believing their partner would be interested in joining such a program [36]. For smoking cessation, patients were sometimes uncertain about involving their family members to be involved in discussions about cessation because they did not want to burden their family, or in contrast because they wanted to maintain personal control over their choices and behavior changes [30]. A combination of being independent of the support of the partner and not wanting to burden them with behavior change will have to be navigated when supporting couples after cancer to adopt a healthy lifestyle. An intervention that explicitly invites partners to be a part of the lifestyle change and that integrates the couple discussing and making plans on how to do this may be an option to tackle these barriers [35].

Interviewing patients and partners separately has its strengths because we can integrate both views with the same importance, but it is also a limitation. Being able to interview and analyze patients and partners as a couple and not as separate individuals might have revealed more dyadic or relationship processes or have made it easier for the patients to talk about the role of their partner in changing. To reach an equal sample of male and female patients, we targeted patients with testicular cancer (only presented in male sex) and breast cancer (mostly presented in female sex). This selection has resulted in a large age difference between testicular and breast cancer patients as Dutch patients with testicular cancer are on average 35 years of age and patients with breast cancer are 60 years of age at diagnosis. The type of behavior patients change and how they value a healthy lifestyle may have been influenced by their age and life phase [37]. In addition, older breast cancer patients were all of female sex and had male partners, whereas all but one younger testicular cancer patient had female partners. A population-based study on age and gender differences in health-related behavior patterns showed that females eat healthier than males, whereas males exercise more than females independent of age [38], although this and other studies do not explicitly explore the effect of age, gender, and their interaction on changes in behavior. We do not rule out that age and sex or their interaction had an influence on the outcomes of this study, although the sample was too small and diverse to explore. An interview study among breast, colon, and prostate cancer survivors focusing on barriers and facilitators to engaging in physical activity and healthy diet following cancer treatment reported that there were no patterns in the themes they found related to age or gender [29].

Hospitals that want to support cancer patients and their partners in making lifestyle changes should adapt their information to fit the specific cancer experience, acknowledge the contradicting feelings towards lifestyle change, and discuss the information several times into survivorship. An explicit role for partners or the larger social network of patients should be integrated to acknowledge their important role and to tackle assumptions that could function as a barrier to change.

## Data Availability

The raw data is comprised of interview recordings, and they are therefore not available on request due to privacy reasons. The coding book from ATLAS.ti can be shared upon request with the corresponding author.
